# 2-Chloro-*N*-(2-chloro­benzo­yl)benzene­sulfonamide

**DOI:** 10.1107/S1600536810012808

**Published:** 2010-04-10

**Authors:** P. A. Suchetan, B. Thimme Gowda, Sabine Foro, Hartmut Fuess

**Affiliations:** a Department of Chemistry, Mangalore University, Mangalagangotri 574 199, Mangalore, India; b Institute of Materials Science, Darmstadt University of Technology, Petersenstrasse 23, D-64287 Darmstadt, Germany

## Abstract

In the structure of the title compound, C_13_H_9_Cl_2_NO_3_S, the N—H bond is *anti* to the C=O bond and the dihedral angle between the two aromatic rings is 76.9 (1)°. In the crystal structure, mol­ecules are linked by N—H⋯O(S) hydrogen bonds to form inversion dimers.

## Related literature

For background literature and similar structures, see: Gowda *et al.* (2009 ▶, 2010*a* ▶,*b* ▶).
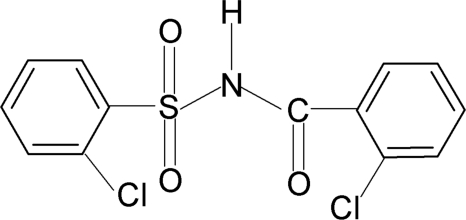


## Experimental

### 

#### Crystal data

C_13_H_9_Cl_2_NO_3_S
*M* *_r_* = 330.17
Monoclinic, 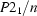

*a* = 6.5943 (6) Å
*b* = 10.9167 (9) Å
*c* = 20.167 (2) Å
β = 95.83 (1)°
*V* = 1444.3 (2) Å^3^
*Z* = 4
Mo *K*α radiation
μ = 0.60 mm^−1^
*T* = 299 K
0.36 × 0.20 × 0.06 mm

#### Data collection

Oxford Xcalibur with a Sapphire CCD detector diffractometer
Absorption correction: multi-scan (*CrysAlis RED*; Oxford Diffraction, 2009 ▶) *T*_min_ = 0.813, *T*_max_ = 0.965
5854 measured reflections
2942 independent reflections
1919 reflections with *I* > 2σ(*I*)
*R*_int_ = 0.025

#### Refinement

*R*[*F* ^2^ > 2σ(*F* ^2^)] = 0.043
*wR*(*F* ^2^) = 0.098
*S* = 1.01
2942 reflections
184 parameters
1 restraint
H atoms treated by a mixture of independent and constrained refinement
Δρ_max_ = 0.23 e Å^−3^
Δρ_min_ = −0.31 e Å^−3^

### 

Data collection: *CrysAlis CCD* (Oxford Diffraction, 2009 ▶); cell refinement: *CrysAlis RED* (Oxford Diffraction, 2009 ▶); data reduction: *CrysAlis RED*; program(s) used to solve structure: *SHELXS97* (Sheldrick, 2008 ▶); program(s) used to refine structure: *SHELXL97* (Sheldrick, 2008 ▶); molecular graphics: *PLATON* (Spek, 2009 ▶); software used to prepare material for publication: *SHELXL97*.

## Supplementary Material

Crystal structure: contains datablocks I, global. DOI: 10.1107/S1600536810012808/bt5238sup1.cif

Structure factors: contains datablocks I. DOI: 10.1107/S1600536810012808/bt5238Isup2.hkl

Additional supplementary materials:  crystallographic information; 3D view; checkCIF report

## Figures and Tables

**Table 1:** Hydrogen-bond geometry (Å, °)

*D*—H⋯*A*	*D*—H	H⋯*A*	*D*⋯*A*	*D*—H⋯*A*
N1—H1N⋯O2^i^	0.85 (1)	2.06 (1)	2.913 (2)	176 (2)

Symmetry code: (i) 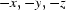.

**Fig. 1.**
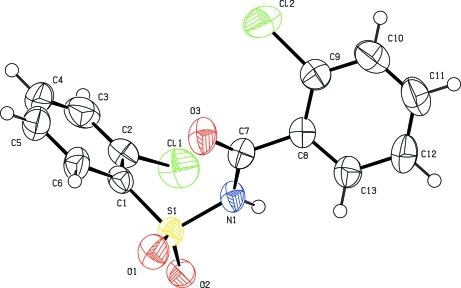
Molecular structure of the title compound, showing the atom-labelling scheme. Displacement ellipsoids are drawn at the 50% probability level.

**Fig. 2.**
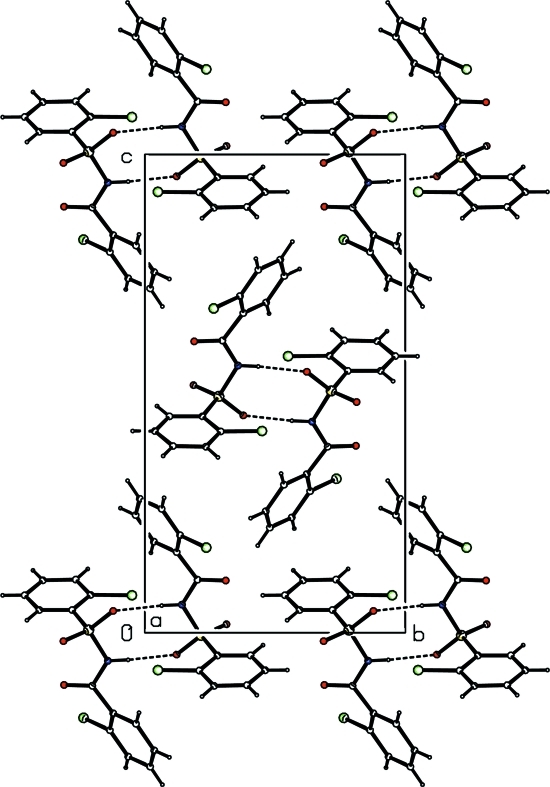
Molecular packing in the title compound. Hydrogen bonds are shown as dashed lines.

## supplementary crystallographic information

### Comment

As a part of studying the effect of ring and the side chain substituents on the crystal structures of *N*-aromatic sulfonamides (Gowda *et al.*, 2009, 2010*a*,*b*), the structure of N-(2-chlorobenzoyl)2-chlorobenzenesulfonamide (I) has been determined. The conformation of the N—H bond in the C—SO_2_—NH—C(O) segment is *anti* to the C═O bond (Fig.1), similar to those observed in *N*-(benzoyl)benzenesulfonamide (II) (Gowda *et al.*, 2009), *N*-(2-chlorobenzoyl)benzenesulfonamide (III)(Gowda *et al.*, 2010*a*) and *N*-(benzoyl)2-chlorobenzenesulfonamide (IV)(Gowda *et al.*, 2010*b*).

Further, the conformation of the C═O bond in the C—SO_2_—NH—C(O) segment of (I) is *syn* to the *ortho*-Cl in the benzoyl ring, similar to that observed in (III).

The molecules are twisted at the S atom with the torsional angle of 66.5 (2)°, compared to those of -66.9 (3)° in (II), -59.0 (2)° (molecule 1) and -67.3 (2)° (molecule 2) in (III), and 66.7 (2)° in (IV).

The dihedral angle between the sulfonyl benzene ring and the —SO_2_—NH—C—O segment is 86.9 (1)°, compared to the values of 86.5(0.1) in (II), 87.3 (1)° (molecule 1) and 73.3 (1)° (molecule 2) in (III), and 87.3 (1)° in (IV). Furthermore, the dihedral angle between the sulfonyl and the benzoyl benzene rings is 76.9 (1)°, compared to the values of 80.3(0.1) in (II), 69.8 (1)° (molecule 1) and 89.8 (1)° (molecule 2) in (III) and 73.3 (1)° in (IV).

The packing of molecules linked by of N—H···O(S) hydrogen bonds(Table 1) is shown in Fig. 2.

### Experimental

The title compound was prepared by refluxing a mixture of 2-chlorobenzoic acid, 2-chlorobenzenesulfonamide and phosphorous oxy chloride for 3 h on a water bath. The resultant mixture was cooled and poured into ice cold water. The solid obtained was filtered, washed thoroughly with water and then dissolved in sodium bicarbonate solution. The compound was later reprecipitated by acidifying the filtered solution with dilute HCl. It was filtered, dried and recrystallized.

Prism like colourless single crystals of the title compound used in X-ray diffraction studies were obtained by slow evaporation of its toluene solution at room temperature.

### Refinement

The H atom of the NH group was located in a difference map and its coordinates were refined with a distance restraint of N—H = 0.86 (1) Å. The other H atoms were positioned with idealized geometry using a riding model with C—H = 0.93 Å. All H atoms were refined with isotropic displacement parameters set to 1.2 times the *U*_eq_ of the parent atom.

### Crystal data

**Table:**

C_13_H_9_Cl_2_NO_3_S	*F*(000) = 672
*M**_r_* = 330.17	*D*_x_ = 1.518 Mg m^−^^3^
Monoclinic, *P*2_1_/*n*	Mo *K*α radiation, λ = 0.71073 Å
Hall symbol: -P 2yn	Cell parameters from 1539 reflections
*a* = 6.5943 (6) Å	θ = 2.8–27.8°
*b* = 10.9167 (9) Å	µ = 0.60 mm^−^^1^
*c* = 20.167 (2) Å	*T* = 299 K
β = 95.83 (1)°	Prism, colourless
*V* = 1444.3 (2) Å^3^	0.36 × 0.20 × 0.06 mm
*Z* = 4	

### Data collection

**Table:**

Oxford Xcalibur with a Sapphire CCD detector diffractometer	2942 independent reflections
Radiation source: fine-focus sealed tube	1919 reflections with *I* > 2σ(*I*)
graphite	*R*_int_ = 0.025
Rotation method data acquisition using ω and phi scans.	θ_max_ = 26.4°, θ_min_ = 2.8°
Absorption correction: multi-scan (*CrysAlis RED*; Oxford Diffraction, 2009)	*h* = −8→8
*T*_min_ = 0.813, *T*_max_ = 0.965	*k* = −10→13
5854 measured reflections	*l* = −10→25

### Refinement

**Table:**

Refinement on *F*^2^	Primary atom site location: structure-invariant direct methods
Least-squares matrix: full	Secondary atom site location: difference Fourier map
*R*[*F*^2^ > 2σ(*F*^2^)] = 0.043	Hydrogen site location: inferred from neighbouring sites
*wR*(*F*^2^) = 0.098	H atoms treated by a mixture of independent and constrained refinement
*S* = 1.01	*w* = 1/[σ^2^(*F*_o_^2^) + (0.0397*P*)^2^ + 0.4268*P*] where *P* = (*F*_o_^2^ + 2*F*_c_^2^)/3
2942 reflections	(Δ/σ)_max_ < 0.001
184 parameters	Δρ_max_ = 0.23 e Å^−^^3^
1 restraint	Δρ_min_ = −0.31 e Å^−^^3^

### Special details

**Table:**

Experimental. CrysAlis RED (Oxford Diffraction, 2009) Empirical absorption correction using spherical harmonics, implemented in SCALE3 ABSPACK scaling algorithm.
Geometry. All esds (except the esd in the dihedral angle between two l.s. planes) are estimated using the full covariance matrix. The cell esds are taken into account individually in the estimation of esds in distances, angles and torsion angles; correlations between esds in cell parameters are only used when they are defined by crystal symmetry. An approximate (isotropic) treatment of cell esds is used for estimating esds involving l.s. planes.
Refinement. Refinement of *F*^2^ against ALL reflections. The weighted *R*-factor wR and goodness of fit *S* are based on *F*^2^, conventional *R*-factors *R* are based on *F*, with *F* set to zero for negative *F*^2^. The threshold expression of *F*^2^ > σ(*F*^2^) is used only for calculating *R*-factors(gt) etc. and is not relevant to the choice of reflections for refinement. *R*-factors based on *F*^2^ are statistically about twice as large as those based on *F*, and *R*- factors based on ALL data will be even larger.

### Fractional atomic coordinates and isotropic or equivalent isotropic displacement parameters (Å^2^)

**Table:**

	*x*	*y*	*z*	*U*_iso_*/*U*_eq_	
C1	0.2149 (4)	0.2794 (2)	−0.04754 (11)	0.0395 (6)	
C2	0.3515 (4)	0.2090 (3)	−0.07907 (13)	0.0489 (7)	
C3	0.5018 (5)	0.2657 (3)	−0.11112 (15)	0.0676 (9)	
H3	0.5918	0.2194	−0.1334	0.081*	
C4	0.5167 (5)	0.3920 (4)	−0.10965 (16)	0.0733 (10)	
H4	0.6193	0.4300	−0.1305	0.088*	
C5	0.3847 (5)	0.4618 (3)	−0.07844 (15)	0.0654 (9)	
H5	0.3963	0.5467	−0.0781	0.078*	
C6	0.2350 (4)	0.4058 (2)	−0.04755 (13)	0.0520 (7)	
H6	0.1447	0.4534	−0.0261	0.062*	
C7	0.2465 (4)	0.2022 (2)	0.11091 (12)	0.0419 (6)	
C8	0.3158 (4)	0.1217 (2)	0.16858 (11)	0.0397 (6)	
C9	0.5063 (4)	0.1350 (2)	0.20376 (13)	0.0508 (7)	
C10	0.5629 (5)	0.0641 (3)	0.25910 (15)	0.0685 (9)	
H10	0.6914	0.0736	0.2821	0.082*	
C11	0.4305 (5)	−0.0199 (3)	0.28004 (16)	0.0708 (9)	
H11	0.4685	−0.0668	0.3178	0.085*	
C12	0.2422 (5)	−0.0358 (3)	0.24604 (14)	0.0629 (8)	
H12	0.1530	−0.0937	0.2604	0.076*	
C13	0.1849 (4)	0.0343 (2)	0.19040 (12)	0.0492 (7)	
H13	0.0570	0.0229	0.1672	0.059*	
N1	0.1344 (3)	0.14241 (17)	0.05872 (10)	0.0417 (5)	
H1N	0.122 (4)	0.0648 (9)	0.0568 (12)	0.050*	
O1	−0.0977 (3)	0.31318 (15)	0.01764 (9)	0.0565 (5)	
O2	−0.0841 (3)	0.12191 (15)	−0.04562 (8)	0.0492 (5)	
O3	0.2790 (3)	0.31054 (16)	0.10914 (9)	0.0573 (5)	
Cl1	0.34230 (13)	0.05007 (7)	−0.07821 (5)	0.0774 (3)	
Cl2	0.68560 (13)	0.23576 (8)	0.17804 (5)	0.0878 (3)	
S1	0.01817 (10)	0.21602 (5)	−0.00569 (3)	0.04066 (18)	

### Atomic displacement parameters (Å^2^)

**Table:**

	*U*^11^	*U*^22^	*U*^33^	*U*^12^	*U*^13^	*U*^23^
C1	0.0459 (14)	0.0355 (14)	0.0351 (13)	0.0001 (11)	−0.0054 (11)	−0.0001 (11)
C2	0.0469 (15)	0.0522 (17)	0.0458 (14)	0.0054 (13)	−0.0033 (13)	−0.0018 (13)
C3	0.0485 (18)	0.100 (3)	0.0544 (18)	0.0085 (18)	0.0058 (15)	0.0070 (18)
C4	0.061 (2)	0.098 (3)	0.059 (2)	−0.025 (2)	−0.0009 (17)	0.032 (2)
C5	0.074 (2)	0.059 (2)	0.0605 (19)	−0.0170 (17)	−0.0039 (18)	0.0159 (16)
C6	0.0678 (18)	0.0376 (15)	0.0497 (16)	−0.0071 (13)	0.0023 (14)	0.0059 (12)
C7	0.0524 (15)	0.0349 (15)	0.0394 (13)	−0.0018 (12)	0.0102 (12)	−0.0042 (12)
C8	0.0510 (15)	0.0342 (14)	0.0346 (13)	0.0022 (12)	0.0071 (12)	−0.0039 (11)
C9	0.0538 (17)	0.0483 (16)	0.0498 (16)	−0.0002 (13)	0.0034 (14)	−0.0035 (13)
C10	0.061 (2)	0.080 (2)	0.062 (2)	0.0072 (18)	−0.0071 (16)	0.0039 (18)
C11	0.080 (2)	0.079 (2)	0.0535 (18)	0.0169 (19)	0.0040 (18)	0.0201 (17)
C12	0.080 (2)	0.0596 (19)	0.0517 (17)	−0.0006 (16)	0.0184 (17)	0.0157 (14)
C13	0.0564 (17)	0.0485 (16)	0.0431 (15)	−0.0011 (13)	0.0069 (13)	0.0024 (13)
N1	0.0602 (14)	0.0242 (10)	0.0394 (11)	−0.0032 (10)	−0.0020 (10)	−0.0021 (9)
O1	0.0614 (12)	0.0370 (10)	0.0730 (13)	0.0118 (9)	0.0154 (10)	0.0003 (9)
O2	0.0528 (11)	0.0335 (9)	0.0577 (11)	−0.0052 (8)	−0.0111 (9)	−0.0007 (8)
O3	0.0882 (15)	0.0323 (10)	0.0499 (11)	−0.0107 (10)	−0.0011 (10)	−0.0042 (9)
Cl1	0.0773 (6)	0.0542 (5)	0.1022 (7)	0.0199 (4)	0.0158 (5)	−0.0171 (4)
Cl2	0.0611 (5)	0.0780 (6)	0.1224 (8)	−0.0201 (4)	0.0001 (5)	0.0151 (5)
S1	0.0464 (4)	0.0281 (3)	0.0469 (4)	0.0016 (3)	0.0015 (3)	0.0005 (3)

### Geometric parameters (Å, °)

**Table:**

C1—C2	1.387 (3)	C8—C9	1.386 (3)
C1—C6	1.387 (3)	C8—C13	1.388 (3)
C1—S1	1.760 (2)	C9—C10	1.378 (4)
C2—C3	1.383 (4)	C9—Cl2	1.733 (3)
C2—Cl1	1.736 (3)	C10—C11	1.362 (4)
C3—C4	1.383 (4)	C10—H10	0.9300
C3—H3	0.9300	C11—C12	1.367 (4)
C4—C5	1.358 (4)	C11—H11	0.9300
C4—H4	0.9300	C12—C13	1.379 (4)
C5—C6	1.364 (4)	C12—H12	0.9300
C5—H5	0.9300	C13—H13	0.9300
C6—H6	0.9300	N1—S1	1.649 (2)
C7—O3	1.203 (3)	N1—H1N	0.852 (10)
C7—N1	1.387 (3)	O1—S1	1.4153 (17)
C7—C8	1.492 (3)	O2—S1	1.4312 (17)
			
C2—C1—C6	119.0 (2)	C10—C9—C8	120.8 (3)
C2—C1—S1	123.2 (2)	C10—C9—Cl2	117.5 (2)
C6—C1—S1	117.8 (2)	C8—C9—Cl2	121.6 (2)
C3—C2—C1	119.7 (3)	C11—C10—C9	120.0 (3)
C3—C2—Cl1	118.7 (2)	C11—C10—H10	120.0
C1—C2—Cl1	121.6 (2)	C9—C10—H10	120.0
C4—C3—C2	119.3 (3)	C10—C11—C12	120.5 (3)
C4—C3—H3	120.3	C10—C11—H11	119.7
C2—C3—H3	120.3	C12—C11—H11	119.7
C5—C4—C3	121.5 (3)	C11—C12—C13	119.8 (3)
C5—C4—H4	119.3	C11—C12—H12	120.1
C3—C4—H4	119.3	C13—C12—H12	120.1
C4—C5—C6	119.2 (3)	C12—C13—C8	120.8 (3)
C4—C5—H5	120.4	C12—C13—H13	119.6
C6—C5—H5	120.4	C8—C13—H13	119.6
C5—C6—C1	121.3 (3)	C7—N1—S1	122.53 (17)
C5—C6—H6	119.3	C7—N1—H1N	123.0 (17)
C1—C6—H6	119.3	S1—N1—H1N	114.4 (17)
O3—C7—N1	121.6 (2)	O1—S1—O2	119.05 (11)
O3—C7—C8	124.1 (2)	O1—S1—N1	109.06 (11)
N1—C7—C8	114.3 (2)	O2—S1—N1	104.36 (10)
C9—C8—C13	118.0 (2)	O1—S1—C1	108.31 (11)
C9—C8—C7	121.8 (2)	O2—S1—C1	109.92 (11)
C13—C8—C7	120.1 (2)	N1—S1—C1	105.26 (11)
			
C6—C1—C2—C3	−1.5 (4)	C8—C9—C10—C11	−0.3 (4)
S1—C1—C2—C3	179.8 (2)	Cl2—C9—C10—C11	−177.6 (2)
C6—C1—C2—Cl1	177.34 (19)	C9—C10—C11—C12	0.9 (5)
S1—C1—C2—Cl1	−1.4 (3)	C10—C11—C12—C13	−0.5 (5)
C1—C2—C3—C4	1.7 (4)	C11—C12—C13—C8	−0.4 (4)
Cl1—C2—C3—C4	−177.1 (2)	C9—C8—C13—C12	0.9 (4)
C2—C3—C4—C5	−1.2 (5)	C7—C8—C13—C12	−176.1 (2)
C3—C4—C5—C6	0.4 (5)	O3—C7—N1—S1	−6.3 (3)
C4—C5—C6—C1	−0.1 (4)	C8—C7—N1—S1	172.07 (17)
C2—C1—C6—C5	0.7 (4)	C7—N1—S1—O1	−49.6 (2)
S1—C1—C6—C5	179.5 (2)	C7—N1—S1—O2	−177.81 (19)
O3—C7—C8—C9	−40.1 (4)	C7—N1—S1—C1	66.4 (2)
N1—C7—C8—C9	141.5 (2)	C2—C1—S1—O1	−177.1 (2)
O3—C7—C8—C13	136.8 (3)	C6—C1—S1—O1	4.1 (2)
N1—C7—C8—C13	−41.5 (3)	C2—C1—S1—O2	−45.5 (2)
C13—C8—C9—C10	−0.6 (4)	C6—C1—S1—O2	135.70 (19)
C7—C8—C9—C10	176.4 (2)	C2—C1—S1—N1	66.3 (2)
C13—C8—C9—Cl2	176.57 (19)	C6—C1—S1—N1	−112.4 (2)
C7—C8—C9—Cl2	−6.4 (3)		

### Hydrogen-bond geometry (Å, °)

**Table:**

*D*—H···*A*	*D*—H	H···*A*	*D*···*A*	*D*—H···*A*
N1—H1N···O2^i^	0.85 (1)	2.06 (1)	2.913 (2)	176 (2)

Symmetry codes: (i) −*x*, −*y*, −*z*.
